# Early systemic sclerosis: short-term disease evolution and factors predicting the development of new manifestations of organ involvement

**DOI:** 10.1186/ar4019

**Published:** 2012-08-17

**Authors:** Gabriele Valentini, Serena Vettori, Giovanna Cuomo, Michele Iudici, Virginia D'Abrosca, Domenico Capocotta, Gianmattia Del Genio, Carlo Santoriello, Domenico Cozzolino

**Affiliations:** 1 Unit of Rheumatology, via Pansini 5, 80131 Naples, Italy; 2 Unit of General Surgery, via Pansini 5, 80131 Naples, Italy; 3 Unit of Respiratory Physiopathology Unit, ASL-SA1, Via Santoriello 2, 84013 Cava De' Tirreni (SA), Italy; 4 Unit of Internal Medicine of the Second University of Napoli, via Pansini 5, 80131 Naples, Italy

## Abstract

**Introduction:**

We investigated early systemic sclerosis (SSc) (that is, Raynaud's phenomenon with SSc marker autoantibodies and/or typical capillaroscopic findings and no manifestations other than puffy fingers or arthritis) versus undifferentiated connective tissue disease (UCTD) to identify predictors of short-term disease evolution.

**Methods:**

Thirty-nine early SSc and 37 UCTD patients were investigated. At baseline, all patients underwent clinical evaluation, B-mode echocardiography, lung function tests and esophageal manometry to detect preclinical alterations of internal organs, and were re-assessed every year. Twenty-one early SSc and 24 UCTD patients, and 25 controls were also investigated for serum endothelial, T-cell and fibroblast activation markers.

**Results:**

At baseline, 48.7% of early SSc and 37.8% of UCTD patients had at least one preclinical functional alteration (*P *> 0.05). Ninety-two percent of early SSc patients developed manifestations consistent with definite SSc (that is, skin sclerosis, digital ulcers/scars, two or more teleangectasias, clinically visible nailfold capillaries, cutaneous calcinosis, X-ray bibasilar lung fibrosis, X-ray esophageal dysmotility, ECG signs of myocardial fibrosis and laboratory signs of renal crisis) within five years versus 17.1% of UCTD patients (*X^2 ^*= 12.26; *P *= 0.0005). Avascular areas (HR = 4.39 95% CI 1.18 to 16.3; *P *= 0.02), increased levels of soluble IL-2 receptor alpha (HR = 4.39; 95% CI 1.03 to 18.6; *P *= 0.03), and of procollagen III aminopropeptide predicted disease evolution (HR = 4.55; 95% CI 1.18 to 17; *P *= 0.04).

**Conclusion:**

Most early SSc but only a few UCTD patients progress to definite SSc within a short-term follow-up. Measurement of circulating markers of T-cell and fibroblast activation might serve to identify early SSc patients who are more likely to develop features of definite SSc.

## Introduction

Raynaud's phenomenon (RP), which occurs in more than 95% of patients affected by systemic sclerosis (SSc), is the most frequent onset manifestation of the disease [1]. The identification of patients with secondary RP, who experience it as the first symptom/sign of SSc or of any other autoimmune systemic rheumatic disease, has long been recognized as a challenge for both the prevention and early treatment of such disorders [2]. Various attempts have been made to address this issue. Fine *et al. *[3] designated "prescleroderma" any condition characterized by RP, digital ischemic changes and SSc marker autoantibodies and/or capillaroscopic findings typical of the scleroderma pattern. Subsequently, LeRoy and Medsger [4] proposed that a condition characterized by RP and either marker autoantibodies or typical capillaroscopic findings be designated "limited" SSc to foster the inclusion of patients not meeting the American College of Rheumatology (ACR) SSc criteria [5] in clinical studies. A few years ago, Koenig *et al. *[6] validated the criteria proposed by LeRoy and Medsger [4] in a large prospective study, and found that RP patients with SSc marker autoantibodies and/or typical SSc capillaroscopic findings and no manifestation other than puffy fingers and/or arthritis, who will be referred to as early SSc patients in the present paper, developed definite SSc in 47%, 69% and 79% of the cases within 5, 10 and 15 years, respectively, from the onset of RP. Here we describe the evolution of the disease in patients with early SSc during a short-term follow-up. Specifically, we looked for baseline factors predictive of the development of further SSc manifestations in early SSc patients. To our knowledge, this topic has not been explored previously.

## Materials and methods

All patients admitted to the Rheumatology Unit of the Second University of Naples for a suspected secondary RP from 1 November 2000 to 31 October 2010 were considered eligible for the study if they fulfilled LeRoy and Medsger's criteria [7] for RP, that is, bilateral, episodic bi- or triphasic color changes of fingers (pallor followed by dusky blueness and/or redness) induced by a cold challenge. Patients who met the ACR criteria for the classification of SSc [4] or any other connective tissue disease were excluded from the study, as were patients displaying any feature consistent with definite SSc [6], that is, skin sclerosis, digital ulcers/scars, two or more teleangectasias, clinically visible nailfold capillaries, cutaneous calcinosis, X-ray bibasilar lung fibrosis, X-ray esophageal dysmotility, ECG signs of myocardial fibrosis (cardiac blocks, Q waves), blood tests (serum creatinine) indicative of previous scleroderma renal crisis.

After giving informed written consent, according to standard clinical practice and to ensure a correct classification, the selected RP patients underwent: a detailed history and physical examination, to identify any of the previously listed clinical signs that excluded enrolment in the study, puffy fingers, and present or previous arthritis; routine laboratory investigations, devoted to exclude comorbidities and not relevant to the definition of the disease subset (that is, early SSc, definite SSc, UCTD), including blood cell count, urinalysis, blood urea nitrogen (BUN), serum creatinine, alanine aminotransferase (ALT), aspartate aminotransferase (AST), erythrosedimentation rate (ESR), serum protein electrophoresis with the evaluation of gammaglobulin concentration, serum C3 and C4 concentration; nailfold videocapillaroscopy (NVC) using an optical probe videocapillaroscope equipped with a ×200 magnification contact lens and connected to image analysis software (Videocap, DS MediGroup, Milan, Italy). The nailfold of the second, third, fourth and fifth finger was examined bilaterally in each patient. Four consecutive fields extending over 1 mm in the middle of the nailfold were studied per finger. The procedure was carried out by a physician (MI) experienced in NVC [8-10]; an autoantibody screening and profiling of sera collected at the first visit, performed as previously described [11], including antinuclear antibodies (ANA), SSc and other connective tissue disease marker autoantibodies, namely anti-Scl-70, anticentromere (ACA), anti-RNA polymerase III, anti-fibrillarin, anti-PmScl, anti-Th/To, anti-SSA, anti-SSB, anti-Sm, anti-Jo1, anti-U1RNP and anti-dsDNA antibodies; chest X-ray, barium esophageal X-ray and ECG to identify patients not eligible for the study because of findings consistent with lung, esophageal or cardiac SSc involvement as assessed by routine examinations and current treatment. Thus, patients satisfying the Koenig *et al. *criteria for early SSc [6], that is, RP plus either SSc marker autoantibodies and/or megacapillaries or avascular areas and no manifestation other than puffy fingers and/or arthritis, were enrolled in the study. In addition, patients who met the criteria for undifferentiated connective tissue disease (UCTD) (ANA positivity, but no SSc marker or any other connective tissue disease autoantibody, no scleroderma videocapillaroscopic findings or any clinical manifestation pathognomonic of any other connective tissue disease) [12,13], were also enrolled in the study.

At baseline, all patients underwent B-mode echocardiography, lung function tests, and esophageal manometry. The detection of diastolic abnormalities at B-mode echocardiography, indicated by an inverted ratio between early (E)/late (*atrial = *A) ventricular filling velocity (E/A ratio < 1), in the absence of arterial hypertension, coronary artery disease and other symptoms/signs of cardiac disease, was regarded as early scleroderma heart involvement [14]. The detection of a diffusing lung capacity for carbon monoxide (DLCO) or a forced vital capacity (FVC) <80% of the predicted values in the absence of a smoking habit and/or obstructive lung disease at lung function study was regarded as SSc lung involvement [15,16]. The detection of a basal low esophageal sphincter (LES) pressure <15 mmHg, with or without impaired peristalsis, at esophageal manometry was regarded as early SSc esophageal involvement [8].

Lastly, 45 out of the enrolled 76 patients (21 early SSc and 24 UCTD patients) and 25 controls, matched for sex and age and affected with osteoarthritis or primary fibromyalgia syndrome, were investigated for serum endothelial, T-cell and fibroblast activation markers, namely soluble E-selectin (sE-selectin); soluble IL-2 receptor alpha (sIL-2Rα); carboxyterminal telopeptide of type I collagen (ICTP), and aminoterminal propeptide of type III collagen (PIIINP). SIL-2Rα and sE-selectin concentrations were measured by a multiplex suspension immunoassay. The assay was based on the use of polystyrene spectrally encoded beads of 5 to 6 μm diameter as the solid support. Each bead was coupled with a capture antibody specific to the analyte of interest. A first incubation step allowed the binding of the analyte from the test sample to the solid support, then the complex bead-capture antibody-analyte was challenged with a biotinylated detection antibody. Finally, a streptavidin-phycoerythrin conjugate, the reporter molecule, was added to the system to complete the reaction on the surface of each bead, thus labeling the analytes (either sIL-2Rα or sE-selectin). The assay was read with a double laser-based instrument (Luminex 200, Luminex Corporation, Austin, Texas, USA), which identifies each bead by a distinct spectral region and quantifies the concentration of the bound analyte (pg/ml) by analyzing the fluorescence intensity of the streptavidin-phycoerythrin conjugate. Capture and detection antibodies directed against sIL-2Rα and sE-selectin were both originated in mice. All reagents for this assay were provided by Merk Millipore, Billerica, MA, USA. ICTP and PIIINP concentrations were measured by a conventional competitive radio-immunoassay (RIA) and expressed as μg/l, using the UniQ kits by Orion Diagnostica, Espoo, Finland. Again, none of the investigated activation markers contributed to the definition of the disease subset (that is, early SSc, definite SSc, UCTD).

Each patient was re-evaluated every six months for symptoms/signs of SSc or any other connective tissue disease, and underwent yearly ECG, chest and esophageal X-ray, and lung function tests. The follow-up status was assessed in December 2011.

The study protocol was reviewed and approved by the local Ethics Committee.

### Statistics

GraphPad Prism 5.0 (GraphPad Software Inc., San Diego, California, U.S.A.) and MedCalc 11.3 (MedCalc Software bvba, Mariakerke, Belgium) for Windows software were used for statistical analyses. Continuous data were expressed as mean ± SD and median with range, and were compared by Student's *t*-test or Mann-Whitney U test as appropriate. Categorical data were analysed by Fisher's exact test. Kaplan-Meier curves were used to describe the cumulative rates of SSc manifestations over time in the subgroups of patients, and the log-rank test was applied to analyse differences. Risk prediction was assessed by univariate and multivariate logistic regression analysis. Receiver-operating characteristic (ROC) curve analysis was performed to identify the cut-off values of both activation markers predicting the evolution of early SSc to definite SSc and their respective sensitivity and specificity. Statistical significance was expressed by a *P*-value < 0.05.

## Results

From 1 November 2000 to 31 October 2010, 76 patients with RP who fulfilled the entry criteria were admitted to the outpatient clinic. The cohort consisted of 74 women and 2 men, aged from 17 to 73 years (median 41 years), with a disease duration from RP onset ranging from 0.5 to 30 years, (median 3.5 years). Thirty-nine (51.3%) of them fulfilled the criteria for early SSc and 37 (48.7%) for UCTD.

Table 1 shows the main epidemiologic, clinical, laboratory and capillaroscopic features of the 39 early SSc and 37 UCTD patients. The two groups were similar in terms of age, sex, RP duration, ANA positivity, prevalence of abnormal routine immune-inflammatory parameters and smoking habit. By definition, serum SSc-marker autoantibodies (36/39 (92.3%) vs 0/37; *P *< 0.0001) and a capillaroscopic scleroderma pattern (23/39 (59%) vs 0/37; *P *< 0.0001) were detected only in early SSc patients. ANA titers ranged from 1:80 to 1:5,120 (median 1:640) in both groups. Arthritis was found only in UCTD patients (4/37 (10.8%) vs 0/39) (*P *= 0.05). Interestingly, in the early SSc group, the two most frequent autoantibody specificities discriminated patients with a significantly different disease duration; as expected, the ACA group (*n *= 26) had a median disease duration of five years (range 1 to 24), whereas the anti-Scl-70 group (*n *= 8) of 1.5 years (range 0.5 to 3) (*P *= 0.007). Disease duration was one year in the patient positive to anti-Th/To and anti-U1-RNP, and one, six and seven years in the three patients with no marker autoantibodies. Neither the prevalence of the capillaroscopic scleroderma pattern nor avascular areas was related to an autoantibody pattern.

**Table 1:** Epidemiologic, clinical, laboratory and capillaroscopic features at presentation of early SSc and UCTD patients

Feature	Early SSc (*n *= 39)	UCTD (*n *= 37)	*P*
Sex: F/M	38/1	36/1	ns
Age; years (median; range)	41 (17 to 73)	38 (18 to 71)	ns
RP duration; years (median; range)	3 (0.5 to 24)	4 (0.5 to 30)	ns
Puffy fingers	4	0	ns
Arthritis	0	4	0.05
ESR >20 mm/h	5	6	ns
Gammaglobulins >1.5 g/dl	3	2	ns
C3 <80 mg/dl or C4 <15 mg/dl	2	3	ns
			
ANA +	38 (97.4)	37 (100)	ns
ANA titre (median, range)	1:640	1:640	ns
			
SSc-marker antibodies +	(1:80 to 1:5,120)	(1:80 to 1:5,120)	< 0.0001
			
ACA +	36 (92.3)	0	< 0.0001
Anti-Scl-70 +	26 (66.7)	0	0.005
Anti-Th/To	8 (20.5)	0	ns
Anti-U1RNP	1 (2.6)	0	ns
	1 (2.6)	0	
			
NVC SSc pattern	23 (59)	0	< 0.0001
Megacapillaries + avascular areas	19 (48.7)	0	< 0.0001
Avascular areas or megacapillaries	4 (10.2)	0	ns
Smoking habit	17 (43.5)	13 (35.1)	

All data are expressed as numbers and percentages (in brackets), except were otherwise indicated. ACA, anticentromere antibodies; ANA, antinuclear antibodies; ESR, erythrosedimentation rate; F, female; M, male; n, number; NVC, nailfold videocapillaroscopy; RP, Raynaud's phenomenon; SSc, systemic sclerosis; UCTD, undifferentiated connective tissue disease

Table 2 shows the functional heart, lung and esophageal abnormalities detected at enrolment. An E/A ratio <1 was detected in 9/31 (29%) early SSc and in 3/26 (11.5%) UCTD patients, but might be due to confounding factors, such as age and hypertension in 7/9 and 2/3 of these patients, respectively. A DLCO <80% of the predicted value was found in 11/39 (28.2%) early SSc and 10/37 (27%) UCTD patients. A reduced basal LES pressure was found in 11/36 (30.6%) early SSc and 4/27 (14.8%) UCTD patients. Therefore, these investigations did not differ significantly between the two groups. However, when considering a DLCO <70% of the predicted value, the cumulative prevalence of any preclinical functional alteration was 18/39 (46.2%) in early SSc and 9/37 (24.3%) in UCTD patients (*P *= 0.057). These results indicate that preclinical, functional alterations of heart, lung and esophagus can be seen both in patients with strictly defined early SSc and in patients with UCTD.

**Table 2:** Preclinical alterations of heart, lung and esophageal function in early SSc and UCTD patients

	Early SSc (*n *= 39)	UCTD (*n *= 37)	*P*
E/A ratio <1*	2/31 (6.5)	1/26 (3.8)	ns
DLCO <80%	11/39 (28.2)	10/37 (27)	ns
FVC <80%	1/39 (2.6)	1/37 (2.7)	ns
Basal LES pressure <15 mmHg	11/36 (30.6)	4/27 (14.8)	ns
One or more functional alteration	19/39 (48.7)	14/37 (37.8)	ns

*not alternatively explained. All data are expressed as numbers and percentages (in brackets). DLCO, diffusing lung capacity for carbon monoxide; E/A ratio <1, inverted ratio between early (E)/late (*atrial = *A) ventricular filling velocity; FVC, forced vital capacity; LES, low esophageal sphincter; SSc, systemic sclerosis; UCTD, undifferentiated connective tissue disease

Table 3 reports the levels of circulating markers of endothelial, T-cell and fibroblast activation detected in the two groups of patients and controls. Median E-selectin levels were higher in patients with UCTD (0.87 pg/ml vs 0.732 pg/ml in early SSc, *P *< 0.05; vs 0.806 pg/ml in controls, *P *= 0.07). Median ICTP levels were higher in patients with early SSc (3.998 μg/l vs 2.86 μg/l in UCTD and 3.21 μg/l in controls, *P *< 0.05 for both). A value exceeding the 95% percentile of those recorded in controls was found in 6/21 (28.6%) patients with early SSc and in 0/24 patients with UCTD as regards ICTP (*P *= 0.007); in 5/21 (23.8%) patients with early SSc and in 2/24 (8.3%) patients with UCTD as regards PIIINP and sIL-2Rα (*P *> 0.05); and in 0/21 patients with early SSc and in 3/24 (12.5%) patients with UCTD as regards sE-selectin (*P *> 0.05). These results suggest that markers of fibroblast activation, as indicated by increased levels of serum ICTP or PIIINP, are already detectable in early SSc patients and differentiate the latter from UCTD patients (11/21 (52.4%) vs 2/24 (8.3%); *P *= 0.006). T-cell activation as assessed by increased sIL-2Rα levels was detected in both early SSc and UCTD patients, while endothelial activation, as assessed by increased E-selectin was found only in UCTD patients. However, the sample size was too small to draw any definite conclusion in this regard.

**Table 3:** Endothelial, T-cell and fibroblast activation markers in early SSc, UCTD patients and controls

Biomarker	Early SSc (*n *= 21)	UCTD (*n *= 24)	Controls† (*n *= 25)
sE-selectin (pg/ml) median (range)	0.732 (0.35 to 1.2)	0.87 (0.32 to 1.99) #	0.806 (0.4 to 1.76)
sIL-2Rα (pg/ml) median (range)	239 (66.7 to 1,002)	346 (14.3 to 931)	301 (17.9 to 807)
ICTP (μg/l) median (range)	3.998 (1.88 to 10.5)*; °	2.86 (1.17 to 4.25)	3.21 (1.39 to 5.27)
PIIINP (μg/l) median (range)	1.491 (0.002 to 5.43)	1.66 (0.01 to 2.98)	1.65 (0.35 to 7.49)

ICTP, carboxyterminal telopeptide of type I collagen; IL-2Rα, interleukin-2 receptor alpha; PIIINP, aminoterminal propeptide of type III collagen; s, soluble; SSc, systemic sclerosis; UCTD, undifferentiated connective tissue disease. † Affected by osteoarthritic or primary fibromyalgia syndrome. * *P *< 0.05 early SSc vs controls;°*P *< 0.05 early SSc vs UCTD; #*P *< 0.05 UCTD vs early SSc (*P *= 0.07 vs controls)

Early SSc patients were monitored for between 1 and 8 years (17 patient-year; median 3 years); UCTD patients were also monitored for between 1 and 8 years (14 patient-year; median 2 years). Fifteen out of 39 (38.5%) early SSc and 20 out of 37 (54.1%) UCTD patients were followed for at least five years. At December 2011, one patient with early SSc had died from pulmonary thromboembolism secondary to deep vein thrombosis; and another early SSc patient was lost to follow-up.

After enrolment, all patients were treated with 100 mg/day acetylsalicylic acid (ASA) and calcium channel blockers (CCB - either nifedipine 20 to 60 mg/day or amlodipine 5 to 10 mg/day) for RP. The four UCTD patients presenting with arthritis were also treated with hydroxychloroquine (HCQ) 6.5 mg/kg/day.

**Figure 1:**
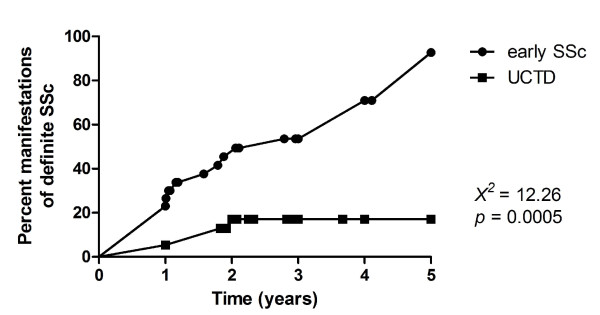
**Time to development of manifestations consistent with definite SSc in early SSc and UCTD patients**. Percent manifestations of definite SSc, as assessed by routine examinations: skin sclerosis, digital ulcers/scars, two or more teleangectasias, X-ray bibasilar lung fibrosis, X-ray esophageal dysmotility, ECG signs of myocardial fibrosis. Time: five years follow-up. Curves were generated using the Kaplan-Meier method and differences between the two groups were analysed by applying the Log-Rank test. SSc, systemic sclerosis; UCTD, undifferentiated connective tissue disease.

Figure 1 shows the time-dependent onset of new manifestations consistent with definite SSc [6] in patients with early SSc and UCTD during follow-up. Nine of the 39 (23.1%) patients with early SSc developed definite SSc at one year, 15/31 (48.4%) at two years, 17/27 (62.9%) at three years, 18/25 (72%) at four years, and 23/25 (92%) at five years from presentation. On the other hand, 2/37 (5.4%) UCTD patients developed definite SSc at one year, and 5/29 (17.1%) at two years (*X*^2 ^= 12.26; *P *= 0.0005). Interestingly, no UCTD patient had developed additional manifestations two years after presentation.

Table 4 shows the various manifestations of definite SSc that developed in early SSc and UCTD patients during the follow-up period. Specifically, digital ulcers/scars developed in 5/39 (12.8%) patients with early SSc and in 1/37 (2.7%) with UCTD; 2 or more telangectasias became apparent in 14/39 (35.9%) patients with early SSc and in 0/37 with UCTD; skin sclerosis developed in 4/39 (10.2%) patients with early SSc (sclerodactyly in 3 cases and skin sclerosis proximal to the elbows in 1) and in 0/37 with UCTD, lung fibrosis as detected by chest X-ray developed in 2/39 (5.1%) patients with early SSc and 1/37 (2.7%) with UCTD, esophageal dysmotility in 3/39 (7.7%) patients with early SSc and 3/37 (8.1%) with UCTD, cardiac blocks in 2/39 (5.1%) patients with early SSc and 0/37 with UCTD. Therefore, considering all the above listed complications, manifestations consistent with definite SSc developed in 30/39 (76.9%) with early SSc and 5/37 (13.5%) patients with UCTD (*P *< 0.0001).

**Table 4:** Manifestations of definite SSc at routine examinations in early SSc and UCTD patients at follow-up

	Early SSc (*n *= 39)					UCTD (*n *= 37)		*P*†
	**1° y**	**2° y**	**3° y**	**4° y**	**5° y**	**1° y**	2° y*	
Digital ulcers/scars	4	5	5	5	5 (12.8)	0	1 (2.7)	
Teleangeactasias (≥2)	7	9	10	10	14 (35.9)	0	0	
Skin sclerosis	0	3	4	4	4 (10.2)	0	0	
mRSS (median, range)	-	2	3.5 (2 to 8)	3.5 (2 to 8)				
Chest X-ray bibasilar lung fibrosis	2	2	2	2	2 (5.1)	0	1 (2.7)	
Esophageal dysmotility at barium X-ray	2	2	2	2	3 (7.7)		3 (8.1)	
Cardiac blocks and/or Q waves	0	0	1	2	2 (5.1)	0	0	
TOTAL					30 (76.9)		5 (13.5)	< 0.0001

All data are expressed as numbers and percentages (in brackets). mRSS, modified Rodnan Skin Score; SSc, systemic sclerosis; UCTD, undifferentiated connective tissue disease; y, year. † statistical difference at the end of the follow-up period; *none of the UCTD patients developed further manifestations of definite SSc after the second year of follow-up.

Finally, we evaluated whether the serologic, capillaroscopic, clinical and functional parameters were predictive of the development of additional clinical and/or functional alterations in patients with early SSc. The HR was significant for PIIINP (HR = 4.55; 95% CI 1.18 to 17; *P *= 0.04), sIL-2Rα (HR = 4.39; 95% CI 1.03 to 18.6; *P *= 0.03), and avascular areas (HR = 4.39; 95% CI 1.18 to 16.3; *P *= 0.02). No factor was associated with the development of additional manifestations in patients with UCTD.

In order to evaluate the cut-off value with higher sensitivity and specificity of each activation marker potentially useful in predicting the development of further SSc manifestations in the short term, we performed a ROC analysis for each marker, but we found inconsistent results for both sIL-2Rα (area under the observed receiver operating curve (AUC) 0.6250) and PIIINP (AUC 0.5515), possibly because of the low number of patients investigated for each marker who did not develop any manifestation during follow-up (4/21 of those evaluated).

## Discussion

The aim of this study was to investigate the disease course in patients with strictly defined early SSc compared to patients with UCTD during the five years after presentation, and to look for baseline features predictive of further organ involvement. The results reported herein confirm, in a larger population, our previous data on the prevalence of preclinical organ involvement in early SSc [11] and demonstrate that preclinical, scleroderma-type, functional heart or lung or esophageal abnormalities are common both in patients with early SSc and in patients with UCTD. Impaired left ventricular filling, as the earliest finding of SSc myocardial disease [17], and/or a reduced DLCO, as the earliest finding of SSc pulmonary involvement [16], and/or a reduced basal LES pressure, as the earliest detectable SSc esophageal abnormality [18], were detected in 19/39 (48.7%) early SSc and in 14/37 (37.8%) UCTD patients (*P *> 0.05). Therefore, both patients with early SSc, who have recently been shown to be at very high risk for developing definite SSc during a long-term observation, that is, 10 and 15 years [6], and patients with UCTD, who have a much lower risk of developing definite SSc, may already have a preclinical scleroderma-like internal organ involvement at presentation. Our results indicate that, at presentation, patients with early SSc do not differ in any aspect from patients with UCTD. Of note, however, a reduced basal LES pressure at enrolment was approximately two-fold more frequently observed in early SSc than in UCTD patients (11/36 - 30.6% versus 4/27 - 14.8%; *P *= 0.23). This suggests that a subclinical scleroderma-like esophageal involvement in early SSc, as compared to UCTD patients, could be statistically significant in a larger cohort study.

The above findings were not entirely unexpected. In various studies of patients with RP, lung function alterations were more prevalent in, but not exclusive to, patients with typical capillaroscopic abnormalities and/or marker autoantibodies [19-26].

We also found that circulating markers of endothelial, and B-cell (gammaglobulins) and T-cell (sIL-2Rα) responses are altered in some patients with early SSc or UCTD, whereas abnormalities in circulating markers of fibroblast activation, namely ICTP ± PIIINP, appear to be restricted to early SSc. However, these findings must be interpreted with caution, due to the small number of our test sera.

We found that patients with early SSc developed manifestations consistent with definite SSc in a significantly higher percentage than patients with UCTD (92% vs 17.1% at five years). Therefore, even preclinical internal organ involvement does not seem to differ in patients with early SSc with respect to those with UCTD, the disease course is quite different. It is noteworthy that the drugs prescribed at admission in patients from both groups (calcium channel blockers, ASA) are not known to influence the disease course.

We also investigated the predictive role of any parameter evaluated at presentation for the development of further scleroderma-like manifestations. We did not find any parameter predictive of further disease manifestations in patients with UCTD. Instead, the presence of avascular areas at nailfold capillaroscopy and of increased serum levels of PIINP and sIL-2Rα was significantly associated with an increased risk of developing definite SSc in patients with early SSc. These results suggest that patients with early SSc should undergo evaluation of fibroblast and T-cell activation markers. Further studies on larger cohorts are required to define the role of these and other potential activation markers, not assessed in our study, in the identification of patients at risk for developing definite SSc.

We detected a higher incidence of definite SSc with respect to Koenig *et al. *[6]. In fact, 47% of their patients with early SSc and 4% of their patients with UCTD had developed definite SSc at five years. This discrepancy probably depends on two factors: first, 93.45% of the patients studied by Koenig and colleagues had been referred by primary care physicians, whereas our Unit is a tertiary referral center; second, the prevalence of anti-Scl-70 positivity has long been known to be much higher in Italian (25%) [27] than in French Canadian (9.7%) patients [10].

Taken together, our results indicate that patients with early SSc or UCTD in whom a scleroderma-like functional internal organ involvement has been detected should be regarded as being affected by SSc and, therefore, challenge the time-honoured practice of calculating the duration of SSc starting from the first non-RP symptom. In addition, from a clinical point of view, our data suggest that internal organ involvement should be assessed by sensitive and specific functional studies in patients presenting with early SSc or UCTD in order to identify any early alteration. Recently, researchers from the European Scleroderma Trials and Research (EUSTAR) group proposed criteria for the very early diagnosis of SSc (VEDOSS) [28], namely RP, puffy fingers, marker autoantibodies and typical capillaroscopic alterations. In this context, based on our findings, we would label "very early SSc" the 20 SSc patients with early SSc who did not display any functional alteration and "early SSc" the 19 patients with RP and any associated functional alteration despite the absence of any sign/symptom. We failed to identify parameters associated with preclinical internal involvement in patients with early SSc or UCTD. Hopefully, the VEDOSS study, which includes a much larger number of patients, will succeed in this task.

## Conclusions

In conclusion, the results of our study should prompt the clinician to investigate early SSc patients for preclinical, functional internal organ involvement and to put them under strict surveillance.

## Abbreviations

ACA: anticentromere antibodies; ACR: American College of Rheumatology; ALT: alanine aminotransferase; ANA: antinuclear antibodies; ASA: acetylsalicylic acid; AST: aspartate aminotransferase; AUC: area under the observed receiver operating curve; BUN: blood urea nitrogen; CCB: calcium channel blockers; CI: confidence interval; DLCO: diffusing lung capacity for carbon monoxide; E/A ratio: early/(A = atrial) late ventricular filling velocity ratio; ECG: electrocardiography; ESR: erythrosedimentaton rate; EUSTAR: European Scleroderma Trials and Research; FVC: forced vital capacity; HCQ. Hydroxychloroquine; HR: hazard ratio; ICTP: carboxyterminal telopeptide of type I collagen; LES: low esophageal sphincter; NVC: nailfold videocapillaroscopy; PIIINP: aminoterminal propeptide of type III collagen; RIA: radio-immunoassay; ROC: receiver-operating characteristic; RP: Raynaud's Phenomenon; SD: standard deviation; sE-selectin: soluble E-selectin;sIL-2Ra: soluble IL-2 receptor alpha; SSc: systemic sclerosis; UCTD: undifferentiated connective tissue disease; VEDOSS: very early diagnosis of systemic sclerosis.

## Competing interests

The authors declare that they have no competing interests.

## Authors' contributions

GV conceived, designed and coordinated the study, and drafted the manuscript. SV performed the multiplex suspension immunoassay, participated in performing statistical analysis, in the drafting and in the critical revision of the manuscript. GC acquired clinical data, performed the statistical analysis, and participated in the design of the study and in drafting the manuscript. MI participated in the acquisition of data and statistical analysis. VD performed the radioimmunosorbent assay and participated in the acquisition of clinical data. DCa participated in the acquisition of data. GD performed esophageal manometry and participated in the design of the study. CS performed lung function tests and participated in the design of the study. DC performed B-mode echocardiography and participated in the design of the study. All authors read and approved the final manuscript.
